# Recent advances in biofabricated gut models to understand the gut-brain axis in neurological diseases

**DOI:** 10.3389/fmedt.2022.931411

**Published:** 2022-09-14

**Authors:** Hohyeon Han, Jinah Jang

**Affiliations:** ^1^ School of Interdisciplinary Bioscience and Bioengineering, Pohang University of Science and Technology (POSTECH), Pohang, South Korea; ^2^ Department of Mechanical Engineering, Pohang University of Science and Technology (POSTECH), Pohang, South Korea; ^3^ Department of Convergence IT Engineering, Pohang University of Science and Technology (POSTECH), Pohang, South Korea; ^4^ Institute of Convergence Science, Yonsei University, Seoul, South Korea

**Keywords:** gut-brain axis, neurological disease, enteroendocrine function, biofabrication, *in vitro* gut models

## Abstract

Increasing evidence has accumulated that gut microbiome dysbiosis could be linked to neurological diseases, including both neurodegenerative and psychiatric diseases. With the high prevalence of neurological diseases, there is an urgent need to elucidate the underlying mechanisms between the microbiome, gut, and brain. However, the standardized animal models for these studies have critical disadvantages for their translation into clinical application, such as limited physiological relevance due to interspecies differences and difficulty interpreting causality from complex systemic interactions. Therefore, alternative *in vitro* gut–brain axis models are highly required to understand their related pathophysiology and set novel therapeutic strategies. In this review, we outline state-of-the-art biofabrication technologies for modeling *in vitro* human intestines. Existing 3D gut models are categorized according to their topographical and anatomical similarities to the native gut. In addition, we deliberate future research directions to develop more functional *in vitro* intestinal models to study the gut–brain axis in neurological diseases rather than simply recreating the morphology.

## Introduction

**Figure 1:**
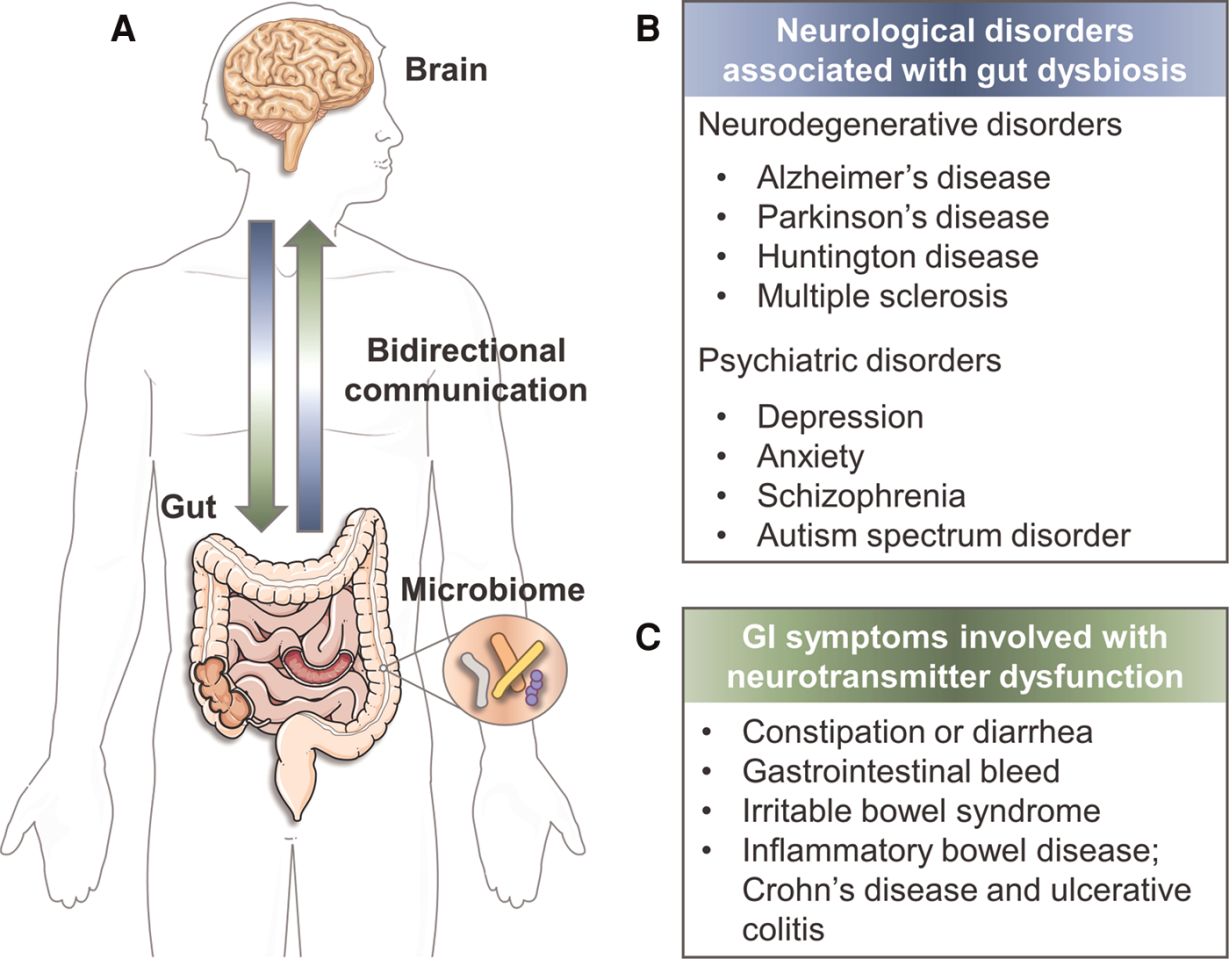
The bidirectional GBA. (**A**) The reciprocal communication between gut and brain. (**B**) Neurological disorders associated with gut dysbiosis (3). (**C**) GI symptoms involved with neurotransmitter dysfunction (4, 5). (created with Servier Medical Art; smart.servier.com).

The gut–brain axis (GBA) refers to bidirectional interactions among the brain, gut, and intestinal microbiome (1, 2) (Figure 1A). Many studies link dysregulation of the GBA to various pathologies from gastrointestinal (GI) symptoms (6) to neurological diseases including neurodegenerative diseases (7–9) and psychiatric disorders (10) (Figure 1B). In particular, the interesting modulation effect of the intestinal microbiome in GBA has been highlighted in neurological diseases such as Alzheimer's disease (11–13), Parkinson's disease (14–16), epilepsy (9, 17), autism spectrum disorders (18, 19) and anxiety or depression (20). Thus far, three major communication pathways have been identified in GBA: (a) the immune system that carries cytokines, (b) the vagus nerve that carries neuronal messages, and (c) the neuroendocrine system that carries neurotransmitters and GI hormones (21).

Conventional animal models for GBA (e.g., gnotobiotic mice) have been invaluable in advancing our insights into how the microbiota and its changes impact the GI and brain (patho-) physiology, while also demonstrating causal linkages between certain microbial cohorts and disease phenotypes (22–25). However, there are critical unsolved issues when translating data from animal models to the human system. First, the interspecies differences in GI topography, microbiome profile, immune system, and brain function limit the relevance of animal models (26). In addition, it is challenging to decipher the etiology of multifactorial disorders involving the GBA due to the extremely convoluted nature of systemic interactions between multiple organs as well as the immune and nervous systems. Furthermore, disentangling the impact of specific microbiome-derived compounds from the context of the whole gut environment is a demanding task (27).

The questionable validity of animal GBA models led to a compelling need for a human-based, preclinical *in vitro* model that is able to dissect the intricate interplay in GBA. From an engineering viewpoint, the essence of *in vitro* GBA models is versatile modularity. In other words, engineers ultimately aim to (a) define the vital factors of complicated disease conditions involving multiple organs, (b) deconvolute them as independent parameters and capture these in the simplest possible configuration, and (c) couple them in combination in a scalable, well-controlled, and reproducible manner (26–28).

In this review, we attempt to suggest research directions to create *in vitro* GBA models in the context of neurological disease as an alternative to conventional animal models. Discussions regarding the *in vitro* brain and blood-brain barrier models for GBA have been extensively described elsewhere (29); therefore, here we would like to delineate the state-of-the-art *in vitro* 3D gut models highlighted in the field of GBA modeling so far. Major biofabrication technologies for gut modeling are classified according to their dimensions and geometrical properties. In addition, we discuss the challenges ahead toward functional gut models for *in vitro* GBA and strategies to surmount them.

## *In vitro* blood-brain barrier models for GBA

**Figure 2:**
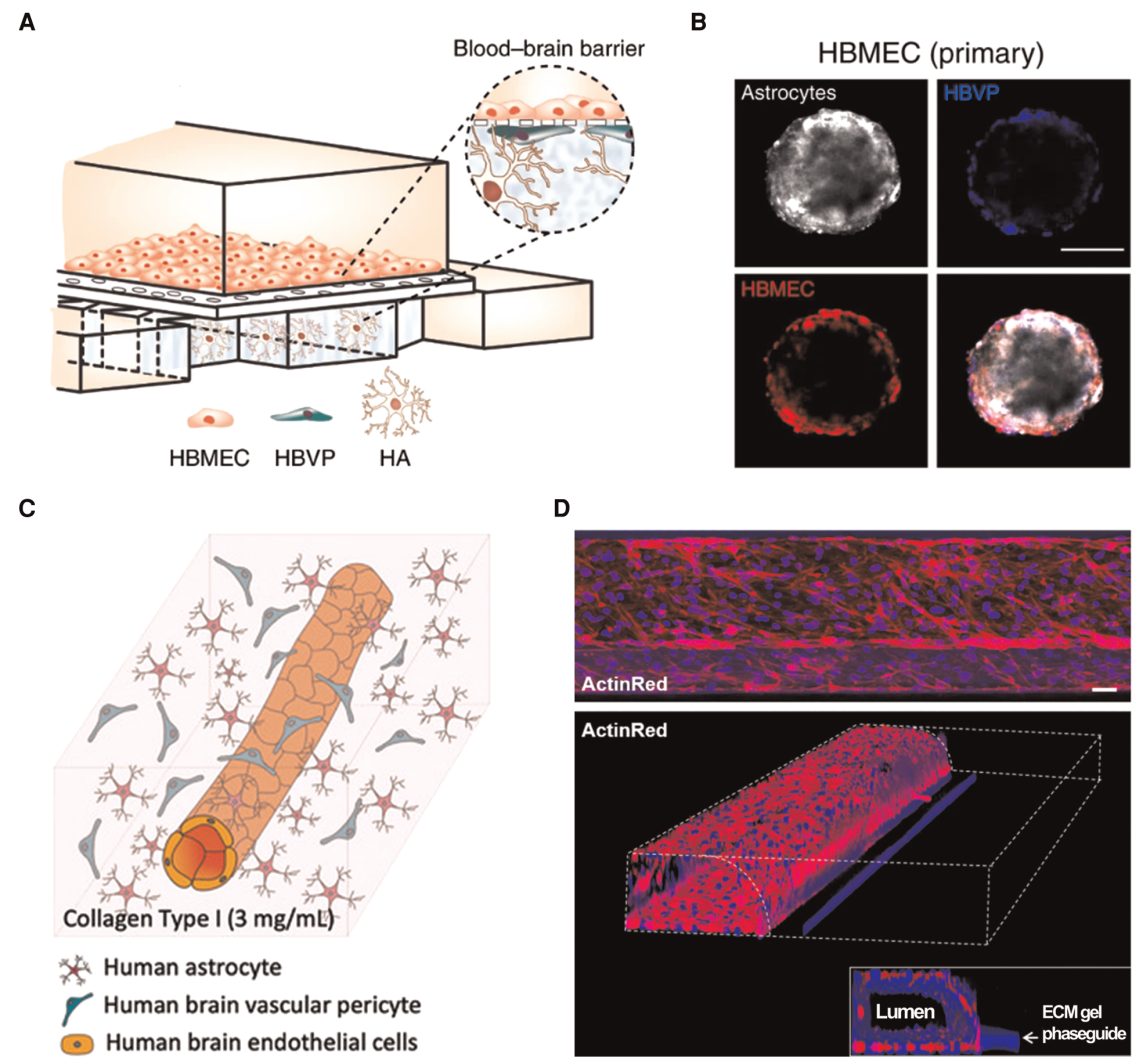
Current *in vitro* blood–brain barrier models. (**A**) Planar MPS based on porous membrane substrate (32). (**B**) Spheroid model (33). (**C**) Perfusable hydrogel-laden MPS (36). (**D**) Perfusable microfluidic model (37). All open access (CC-BY).

Tardy progress in therapeutics development for neurological diseases has driven a need for *in vitro* blood-brain barrier (BBB) models. Based on their geometrical and dimensional features, these models can be categorized as: planar microphysiological systems (MPSs) based on porous membrane substrates (30–32), spheroid models (33, 34), perfusable hydrogel-laden MPS (35, 36), and perfusable microfluidic model (37) (Figure 2). Although these models have provided mechanistic knowledge about neurological diseases, they need to be optimized for future use in GBA platforms. Simplifying the multi-step, complex fabrication process of current BBB models (38, 39) will increase fabrication efficiency of multi-organ platforms, such as GBA. More realistic recapitulation of BBB anatomies (e.g., tubular architectures with curves or bifurcations in various diameters) is indispensable as it enhances the physiological relevance of neuroinflammatory responses in *in vitro* BBB models (40, 41) that will increase the reliability of the GBA models. Finally, free-standing BBB models would be easy to assembly with other organ modules and study the molecular transport between them. For more information about *in vitro* BBB models, the readers may refer to the references (42, 43).

## Current *in vitro* gut models with different anatomical complexity

2D models (e.g. planar cell culture) have been the mainstream approach to studying human intestinal normal- or patho-physiology and defining potential therapeutic strategies (44–46). However, these simplified 2D models cannot truly capture the complexity of intestinal tissue morphology and physiology (46–48); cells interact with their surrounding cells and heterogeneous and complex environments *via* elaborate biochemical signals cascade (49), i.e. cell–cell and cell–matrix interactions. Accordingly, more sophisticated 3D models that closely capture the *in vivo* milieu are believed to bridge the gap between conventional cell cultures and animal models (50–52). Here, we distinguished the up-to-date *in vitro* gut models by their level of anatomical complexity and briefly introduced biofabrication techniques and their features employed to fabricate the models.

### Quasi-3D intestinal epithelium

#### Microphysiological systems

**Figure 3:**
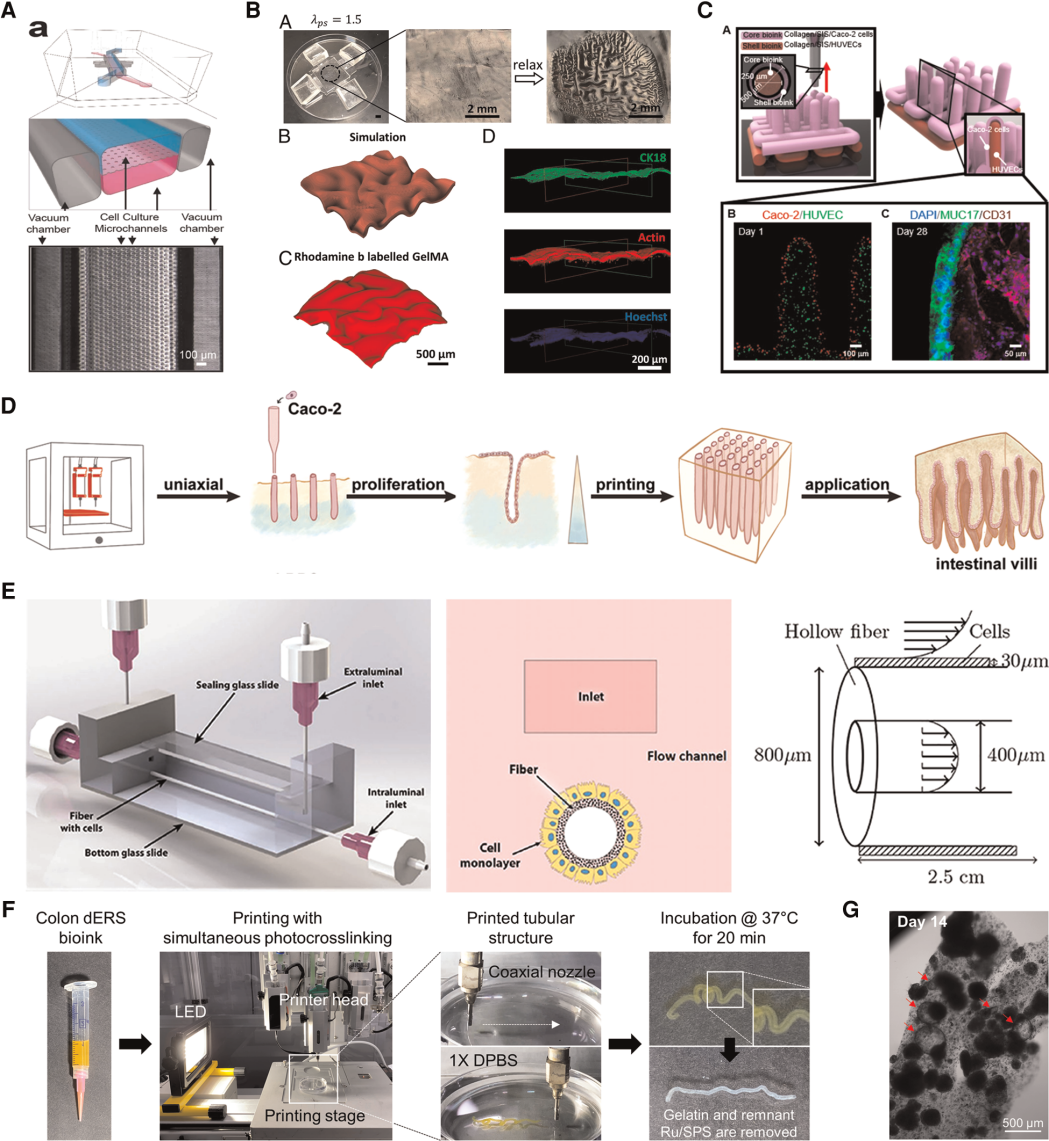
Biofrbrication technologies for *in vitro* gut models. (**A**) Quasi-3D microphysiological systems. Reproduced from Kasendra et al. (56). Open access (CC-BY). (**B**) 3D topography patterning. Reproduced from Chan et al. (57). with permission from the author. (**C**) 3D direct bioprinting. Reproduced from Kim et al. (58). Open access (CC-BY 4.0). (**D**) 3D embedded bioprinting. Adapted from Lian et al. (59) with permission from the publisher. (**E**) 3D hollow scaffold. Reproduced from Langerak et al. (60). Open access (CC-BY 4.0). (**F,G**) 3D coaxial bioprinting assisted with photocrosslinking. Reproduced from Han et al. (61) with permission from the publisher.

Kim et al. (53) opened a window into the world of human intestinal MPSs with their revolutionary work on dynamic mechanical stimulation of the intestinal epithelium. MPSs are defined as microfluidic platform devices that emulate *in vivo* organ physiology and function *in vitro* in a controlled and standardized manner (Figure 3A) (54, 55). The gut-on-a-chip developed by Kim et al. consisted of two parallel microchannels separated by a porous membrane coated with ECM and lined with human enterocyte cell line Caco-2 to reproduce the intestinal barrier. This gut-on-a-chip emulating peristalsis-like motions and luminal flow *in vivo* demonstrated its capability in coculture with the microbiome (62, 63), modeling gut inflammation (64, 65) and intestinal morphogenesis (66, 67) and integration with an ECM membrane (68) and intestinal organoids/enteroids (56, 67, 69) in separate reports. The organoid-mounted microfluidics could be a useful tool for studying dynamic GI hormone secretion related to digestion and response to nutrients (56). Besides, gut-on-a-chip could be a great experimental model for the real-time, non-invasive monitoring of oxygen gradient (63) or mucus production (70).

However, this design only includes part of the four layers of the intestinal walls, resulting in the absence of other elements that can be involved in certain disorders. (46). A fundamental limitation in the molding-and-replication-based fabrication technique is that it not only involves multiple labor-intensive steps but also impedes dimensional expansion into 3D architectures, confining them to mainly 2D or 2.5D geometries (54). Lastly, polydimethylsiloxane (PDMS) is the most favorable material for manufacturing MPSs but the PDMS surface in microfluidic channels also has potential problems in adsorbing small and hydrophobic molecules.

### 3D Intestinal topographies

#### Patterning

Capturing the 3D topography of the intestine in an *in vitro* culture environment can stimulate the exhibition of more realistic functions since numerous signaling gradients are present along the crypt–villus axis (71, 72). Sung et al. used PDMS replica to microfabricate a 3D hydrogel intestinal villi structure (73). Natural and synthetic hydrogels, collagen, and polyethylene glycol diacrylate respectively, were used to make the villi substrate where Caco-2 cells were seeded. The substrate was fully covered with epithelial cells three weeks after seeding and generated similar finger-like epithelial morphologies to those of the human jejunum. This kind of biomimetic scaffolds with a crypt–villus topography can impose geometric boundary conditions that resemble those *in vivo* to organoids or enteroids and guide their self-organization, thus improving their architecture and size (74, 75). Recently, a microfabricated array of collagen crypts and villi cultured with human enteroids effectively generated a self-renewing monolayer, crypt–villus architecture, and opposing gradients of morphogens seen in the native niche of the intestinal epithelium (76).

Another surface feature of the intestine, the circular folds in the mucosal layer, also has certain important roles. The morphological patterns seen in the mucosa considerably increase the surface area, which is beneficial for absorbing nutrients and water (77). In addition, the folded mucosa ease the passage of luminal contents because the luminal diameter of the organ can expand significantly by releasing the folds without causing high stress that may harm the mucosal layer (78–80). In nature, these wrinkle-like structures are generated due to heterogeneous growth rates during development, which generates stress mismatches between tissue layers and accordingly induces mechanical instability (81). Zhao et al. (57) reported a method to recapitulate the folding of artificial mucosa in a controllable manner using layered hydrogel systems (Figure 3B). First, a tough and stretchable hydrogel substrate was made simply by pressing the pregel solution between two layers of glass. PDMS holders stretched and fixed the substrate in a uniaxial or biaxial direction and then an epithelial cell-laden soft gelatin methacrylate (GelMA) hydrogel was attached to the prestretched substrate. As a result, the relaxation of prestretched tough hydrogel induced programmed self-folding. This system demonstrated the mesenchymal condensation process *in vitro*, which facilitates the understanding of mucosal folding.

#### Bioprinting

3D bioprinting is a groundbreaking technology that encompasses a wide range of disciplines and is one of the most attractive tools in tissue engineering (TE) (82, 83). Traditionally, this additive manufacturing process can be classified based on its distinct approach to create a solidified layer: vat polymerization (84), material extrusion (85), and material jetting (86). 3D bioprinting using hydrogel bioink with cells in TE applications is categorized into four types according to their ink-dispensing method: laser-assisted printing, stereolithography, inkjet printing, and microextrusion printing (87). Harnessing bioprinting has established a new pillar of engineering intestinal tissues *in vitro* (58, 59, 61, 88, 89). Lian et al. (59) described an embedded bioprinting strategy using a dual-layered support base to produce a vertical array of intestinal villi with dimensions close to the native tissue (100–200 µm in diameter and ≈2 mm in length) (Figure 3D) (90). Embedded bioprinting enables the extrusion of inks into a (sacrificial) support bath/matrix and is advantageous when using mechanically weak bioinks, which often pose a trade-off between printing fidelity and biocompatibility (91–93). The authors printed a bioink composed of gelatin and Caco-2 cells into a vertical filament shape into GelMA support, which has an upper and lower layer of different concentrations. Gelatin dissolved away at physiological temperature conditions during culture and left microchannel-like villus while still supporting the attachment and growth of encapsulated cells. Intriguingly, the heterogeneous microenvironment of GelMA created gradients in both nutrients and oxygen along the length of the hollow villus structure, simply by reconstructing the hypoxic crypt and normoxic villus bases.

Among various 3D bioprinting techniques, coaxial extrusion printing has provoked tremendous interest because of its ability to directly fabricate perfusable tubular structures by co-extruding multiple materials through a concentrically assembled core–shell nozzle (94, 95). Kim et al. (58) demonstrated a free-standing 3D villi structure composed of a small intestinal epithelium core and microvascular shell using coaxial printing (Figure 3C). To make the projective finger-like villus using collagen/decellularized small intestinal submucosa-based bioink with limited mechanical stiffness, tannic acid was adopted as a crosslinking agent. Analysis of the bioengineered villus showed enhanced cellular activities inclusive of cell viability, proliferation, and the expression of mucin and junctional protein.

### 3D Geometry with a hollow lumen

Although tubular structures of epithelial organs have been of great interest to TE researchers (72, 96–105), there have only been a few studies on the subject of culturing intestinal epithelial cells (IECs) on the inner surface of the hollow structure until recently. Unique advantages that such hollow tubular shapes can offer in intestinal models include (a) accessibility to both the apical and basolateral side of the intestinal epithelium, which is necessary to study trans-epithelial transport (106); (b) extending the lifespan of the intestinal tissue model *via* perfusion through the lumen (107); (c) intraluminal oxygen gradient, which is critical in the mutual interaction between intestinal microbiomes and altered epithelium condition (108); (d) accelerated differentiation of IECs (61, 71). In this regard, subsequent sections explore representative fabrication methods for 3D hollow tubular intestinal models.

#### Conventional scaffold approach

As one of the earliest works on the effect of 3D lumen configuration in intestinal models, a culture system using porous hollow fibers of polyethersulfone was applied to grow Caco-2 cells (71). Differentiation of seeded Caco-2 cells was accelerated over six days. In addition, it was investigated that the tight paracellular barriers formation and brush border enzymes expression was increased compared to the conventional Transwell culture. The shortened time required to differentiate the Caco-2 cells in the hollow tubular system is highly relevant to the rapid differentiation of enterocytes in *in vivo* human intestines (3–5 days) (109, 110).

Microenvironmental cues such as ECM and external forces are important factors to mimic the architecture and physiological parameters of the native intestine (Figure 3E) (60, 106). In this bioengineered intestinal tubule, Caco-2 cells were grown on a human collagen IV- and levodopa-coated hollow fiber membrane with different curvatures (106). In addition, the intestinal tubules were exposed to unidirectional shear stress for the last few days of culture. Under the dynamic condition, Caco-2 cells rapidly formed a monolayer, increasing the speed of the polarization process, inducing apical and basolateral sides, and promoting differentiation into multiple phenotypes including enterocytes, goblet, Paneth, enteroendocrine (EEC), and stem cells compared to the static condition.

Different cell types can be involved by adding layers hierarchically. Roh et al. (111) fabricated silk scaffolds with a hollow lumen space in different sizes using cylindrical molds. Human colonic organoids (colonoids) were seeded on the inner surface of the smaller scaffold and then assembled with the outer scaffold where human primary macrophages were cultured. In response to inflammation caused by Escherichia coli lipopolysaccharide, the migration of macrophages toward the epithelium was observed. Other inflammatory responses, such as increased macrophage infiltration and the production of pro-inflammatory cytokines were also verified.

#### Bioprinting with a high cell population

One current trend in TE is the scaffold-free approach because scaffolding material often interferes with cell-to-cell or cell-to-matrix interactions (112–114). In this context, researchers seek to reinforce cells' ability to produce a matrix by applying proper exogenous stimuli such as stiffness, mechanical stretch, and contact with ECM. These exogenous cues can initiate cellular self-assembly and self-organization when involving external forces such as centrifugation or 3D bioprinting (115). Recently, bioprinting-assisted tissue emergence (BATE) was suggested based on depositioning high-density organoid-forming stem cells directly into a highly permissive ECMs suspension of Matrigel and collagen (88). BATE permitted spatiotemporal control of the cells and the bioactive ECM liquid precursor facilitated cellular self-assembly into macrostructures following the geometrical constraints imposed by the printing process. The printed intestinal organoids robustly fused and evolved into native-like tubular tissue with budding structure, demonstrating their potential in guiding tissue morphogenesis. BATE eminently exemplified the advantage of the automated 3D bioprinting system in handling organoids/enteroids; it can handle these delicate cells in a scalable and reproducible manner (116).

#### Bioprinting with tube-like geometry

The first attempt to fabricate a hollow tubular *in vitro* human intestinal model with the coaxial printing technique was achieved by Kang et al. (89) using a colon-derived decellularized extracellular matrix (dECM)-based bioink. The tissue-specific bioink was supplemented with visible light photoinitiator ruthenium/sodium persulfate (Ru/SPS) to increase the capacity of the crosslinking speed (dERS) (117). Introducing a photoinitiator into the hydrogel bioink is ingenious because of the indispensable requirement for successful coaxial printing that bioinks should be gelated immediately after being extruded from the nozzles (94, 95, 118). Combining tissue-specific microenvironmental niche material and adequate manufacturing methods promoted tissue functionality and printing fidelity simultaneously. The tubular intestinal model printed with Caco-2-laden dERS showed luminal lining of mucin similar to *in vivo*, where a mucus layer covers the intestinal epithelium to house microbiomes and restrain their translocation into underlying tissues (119). Given that *in vitro* models with Caco-2 often fail to develop a luminal mucus layer, the recapitulation of the luminal mucus lining in the colon dERS tubular models is compelling evidence for the significance of combining suitable materials and fabrication techniques.

This approach was further developed by Han et al. (61) to enhance the intestine-specific functions of the model based on the dERS bioink (Figure 3F). The hollow tubular intestinal model was fabricated again with Caco-2 and colon dERS but some important printing parameters (e.g., initial cell density, bioink, and photoinitiator concentration) were fine-tuned. As a result, the single cells that were evenly distributed along the printed tube simultaneously aggregated to form multicellular spheroids and self-organized into lumenized cysts (Figure 3G). This transition—called lumenogenesis—is a hallmark of distinct epithelial morphogenesis that occurs under biomimetic conditions (120). In addition, the differentiation of Caco-2 cells into functional intestinal phenotypes was identified by the expression of EEC markers such as chromogranin A and lysozyme. This indicates that 3D bioprinting and tissue-specific biochemical cues hold promise for geometrical guidance and the accelerated differentiation of accommodated cells.

## The necessity of neuroendocrine models

The gut contributes to the GBA as the body's largest endocrine organ. In particular, EEC cells in the intestinal epithelium produce numerous hormones and neuroactive peptides. These signaling mediators secreted from EEC cells bind to the receptors of the vagus nerve, accomplishing direct bidirectional communication (7). In other words, EEC cells have a paramount role in the neuroendocrine pathway in GBA. However, as discussed in the previous section, the most recent technologies developed to fabricate 3D *in vitro* gut models were focused on recreating the morphological features of the intestine rather than its endocrine function. Likewise, current *in vitro* multi-organ GBA models lack the intrinsic secretory property of the intestine. For example, to elicit mutual responses between the gut and brain, modulation by exogenous immune cells (27) or microbial byproducts (121) were exploited but regulation *via* GI hormones or neurotransmitters has not yet been successfully recapitulated.

An *in vitro* neuroendocrine gut model would be beneficial for studying neurological diseases, considering the crucial functions of EEC hormones and neurotransmitters in GBA. For instance, serotonin is a critical signaling regulator in GBA and about 95% of it is produced by enterochromaffin cells (one phenotype of EEC cells) in the epithelium (122). Serotonin dysfunction is heavily associated with important brain functions such as mood, sleep, and behavior (123). Unfortunately, the fabrication of an *in vitro* EEC model is still in its infancy. In this regard, EEC cell sources are briefly presented as an important component for an *in vitro* neuroendocrine model.

### Candidate cell sources for EEC models

There have been enormous efforts to reconstruct the *in vitro* EEC function at a cellular level. For decades, two immortalized EEC cell lines of human origin, NCI-H716 and HuTo-80, have provided a starting framework to study the secretion of gut hormones *in vitro*. NCI-H716, a poorly differentiated adenocarcinoma of the human cecum (124), is a representative type of distal L-cells among various subtypes of EEC cells. This cell line displays endocrine features including secretory granules and chromogranin A (125) and can secrete GI hormones in response to nutrients (126). Moreover, NCI-H716 exhibits receptors for several neurotransmitters such as gastrin, serotonin, and somatostatin (127). HuTu-80 is derived from duodenal carcinoma and is the only widely available human-derived small intestinal small cell line (128). This cell line also resembles L-cells and has been utilized as a model to study the secretion of tastant-induced gut hormones (129, 130). However, it is difficult to extrapolate the physiological function of the EEC system from single-cell cultures because they lack other types of IECs that influence the production and secretion of gut hormones.

Reimann et al. (131) established a protocol to culture primary enteroendocrine cells *in vitro* and enabled the study of the secretory mechanisms of gut hormones at the molecular level. Primary cells refer to non-transformed *ex vivo* cells that are isolated from tissue specimens obtained during biopsies or surgeries (132). Studies with purified primary human EEC cells enable a better understanding of hormone secretion and the metabolic pathway of the gut (133–135). However, some critical issues remain regarding primary cells; the finite lifespan and proliferation of primary cells should be taken into consideration (136). Relatively small proportions of EEC cells (1% of the intestinal epithelium) are also a challenge when deciphering the dynamics of hormone secretion (137).

**Figure 4:**
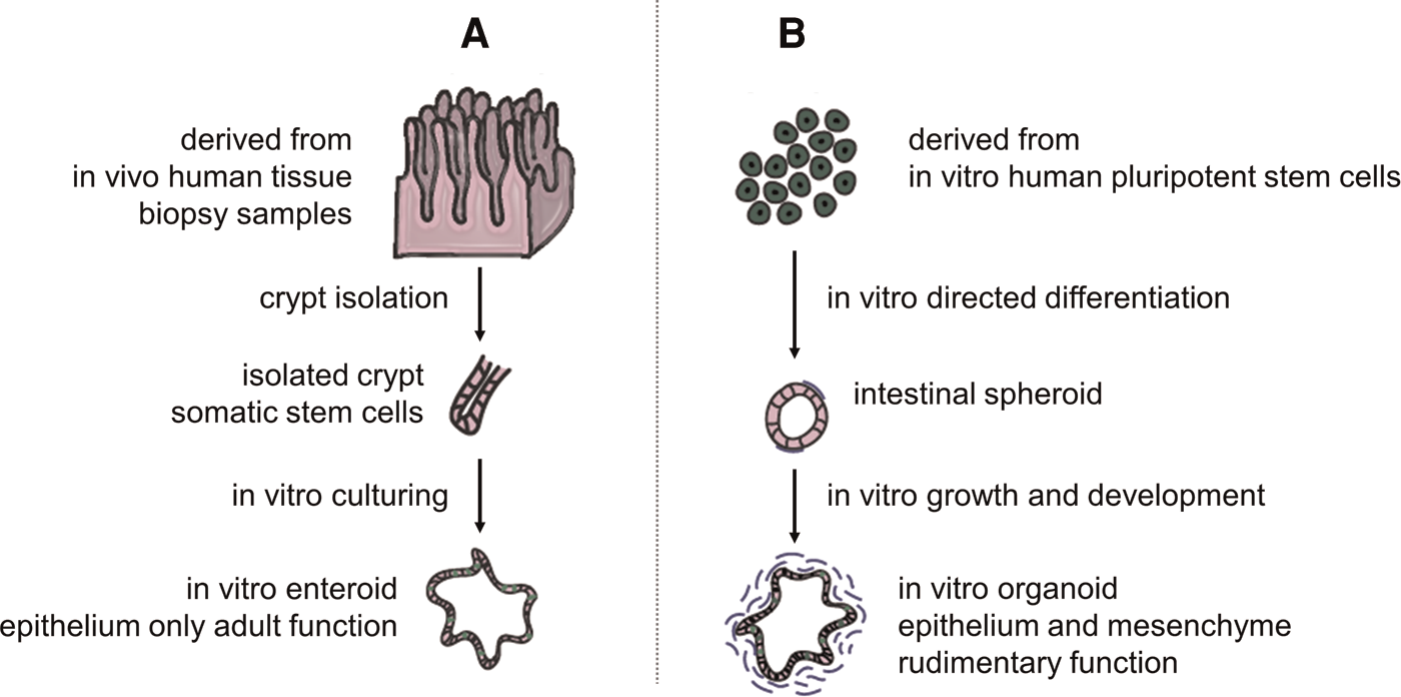
Difference between enteroids and organoids. (**A**) Enteroids are human mini-guts resembling the native intestine derived from adult ISCs of isolated crypts in intestinal biopsy samples. **(B)** Alternatively, intestinal organoids can be directly generated from induced pluripotent stem cells. The terminology, enteroids and organoids, was suggested to differentiate epithelial only *in vitro* cultures from both epithelial and mesenchymal ones, respectively. Adapted from Singh et al. (139). Open access (CC-BY 4.0).

Contrary to primary IECs, intestinal enteroids/organoids maintain their viability for over a year *in vitro*; this indefinite proliferation feature is valuable to studying long-term intestinal illnesses. Identification of adult intestinal stem cells (ISCs) and their niche and generation of an organotypic culture system have engendered advancements in the field of intestinal epithelial study (138) (Figure 4). Interestingly, EEC cells in enteroids and organoids can be enriched by the expression of some translational factors and small molecules (140–143), which is promising for the customization of EEC cells in enteroids/organoids. Intestinal enteroids/organoids have been used in various applications including modeling intestinal development, physiology and pathophysiology, nutrition transport, and metabolism (144). The practical problems associated with enteroids/organoids models is that they are not cost-effective and are difficult to scale up to meet the size requirements of drug screening or TE approaches, where centimeter-scale material is often desired (145, 146). Besides, the microanatomy of organoids/enteroids is typically confined to spheroidal shapes, which fail to recapitulate *in vivo*-like crypt–villus architecture.

## The usefulness of microenvironmental cues in gut models

Imposing microenvironmental cues *via* surrounding material can maneuver the differentiation of EEC cells *in vitro*. For example, Bruïne et al. (127) reported that ECM has a determinant role in the endocrine differentiation of NCI-H716 cells. Culturing NCI-H716 on various extracted ECM components (e.g., colon ECM or collagen IV/heparan sulfate proteoglycan mix) enhances the adhesion of cells and induced EEC differentiation. Of note, individual ECM components, unlike complex combinations, do not induce endocrine differentiation, and the adhesion of cells onto a substrate is a prerequisite for inducing endocrine phenotype (125). Therefore, appropriate ECM environments are paramount for the differentiation of IECs.

As shown in BATE, bioprinting assisted by a proper ECM environment can achieve organoids/enteroids-derived tissue structures at centimeter scale, which is physiologically relevant for use in implantable regenerative medicine. Conventionally, the size of single organoids/enteroids is typically limited to millimeter-scale at best because the inner core becomes deficient in nutrient supply as the organoids/enteroids grow larger, resulting in necrosis (145). Furthermore, bioprinting employing multiple biomaterials is expected to assist in the integration of various organoids/enteroids and enhance their functionality in a single tissue system (147). Additionally, an enteroids polarity reversal strategy was developed based on understanding how basement membrane extract (BME) affects the epithelial polarity. Basolateral-out enteroids grown within BME showed an inversion of polarity when transferred to a BME-free suspension culture environment, enabling easy access to the apical side of the enteroids without technically demanding microinjection. Thus far, many aspects of soluble niche components important for culturing enteroids/organoids have been unraveled, whereas the role of insoluble ECM as a vital niche element remains a mystery (148). Since the IECs interact constantly with the local niche, which is comprised of both soluble factors and ECM gradients, the delicate balance between proliferating and differentiating ISCs is elicited by the dynamic microenvironment along the crypt–villus axis (149).

Compared to single ECMs (e.g. collagen or fibronectin), utilizing dECMs derived from normal or diseased tissue could contribute to mimicking a reliable microenvironment because they could reflect the structural and compositional disorganization of ECM during disease progression (150, 151). Interestingly, Alfano et al. (152) showed different intestinal models using three types of dECM substrates derived from healthy, perilesional, and colorectal carcinoma (CRC) human tissue. The specific characteristics of various cancer cells such as invasive phenotype, turnover, differentiation, and polarization were sustained, recapitulating the native tissue homeostasis and tumorigenesis more faithfully *in vitro*. Further investigations were taken to unveil the biochemical and mechanical features of the three dECMs and their underlying mechanisms regarding increased stiffness in the perilesional and CRC tissue (153). A recent study presented a CRC model that mimics the alteration of ECM in each tumor stage by using a dECM substrate originating from human CRC tissue of different stages (154). As the tumor stage increased, the imbalance of ECM composition was observed similar to *in vivo*, which induced changes in the proliferation and migration of the seeded cancer cells.

## Evaluation techniques for GBA

Although *In vitro* BBB and gut models have seen tremendous progress in recent years, unresolved issues concerning connecting each component into a single GBA model remain. First, most of the state-of-the-art BBB and gut models are fabricated through a multi-step, complicated process that demands a lot of time and effort. In addition, as each model is advanced, they become to include various cellular compositions, making it difficult to find an optimized condition to coculture them. Last but not least, there is no defined method to track the dynamics which occur in *in vitro* GBA models. Many studies rely on visual assessment of cell morphology or end-point analysis since it is challenging to monitor the changes continuously without terminating the sample. However, as the BBB and gut epithelium works as physiological barriers in our body, it is necessary to quantitatively evaluate their wall tightness and integrity *in vitro* in a real-time and non-destructive way.

Trans-epithelial electrical resistance (TEER) is the most representative technique to measure barrier integrity (155). Recently, TEER-interfaced BBB (31, 156, 157) and gut (158) models enabled continuous and non-invasive detection of their barrier properties *in situ* and demonstrated the strength of sensor-implemented tissue platforms. Nevertheless, the above-mentioned models are still confined to a planar dimension and need to be expanded to 3D tissues. So far, only few studies have incorporated electronics into 3D BBB or gut tissue models (159). Moreover, electrochemical biosensors-assisted platforms for monitoring cell secretomes and behavior (160–164) would help chronological and rapid readout of multi-organ axies such as GBA.

## Conclusion

**Figure 5:**
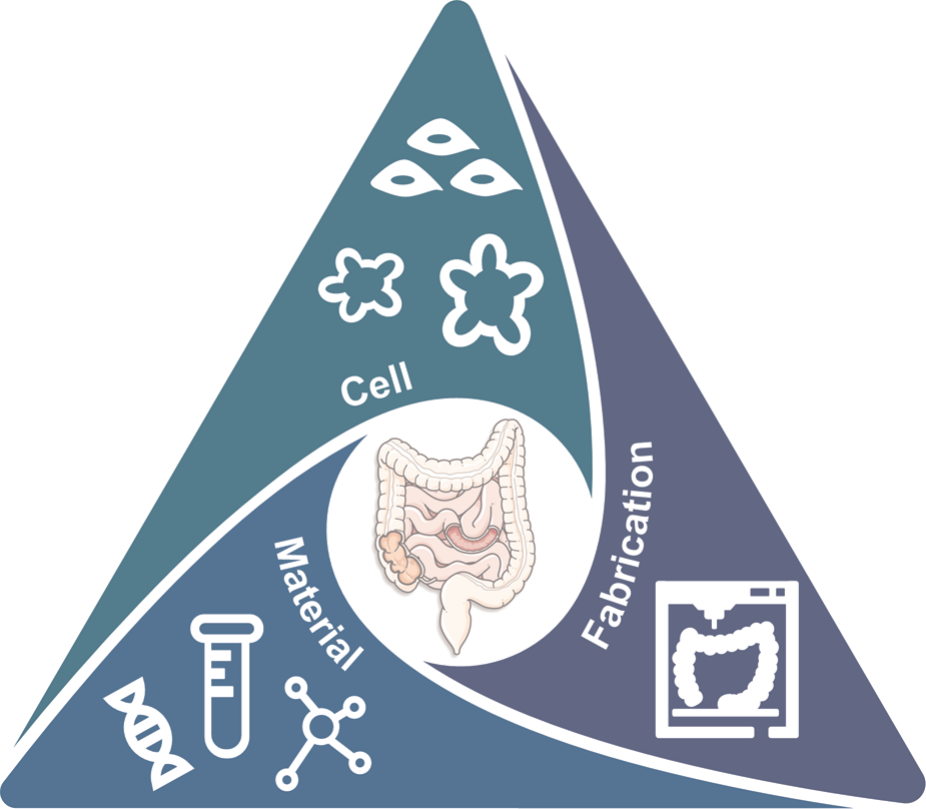
The TE trinity toward advanced *in vitro* gut model for GBA: cell, material, and fabrication.

This paper comprehensively reviews the necessity of EEC models in the future in terms of an *in vitro* tool to unravel the underlying influence of GBA in neurological diseases. The traditional GBA animal models have widened our understanding of the reciprocal interaction between the microbiome, gut, and brain. However, their intrinsic differences in tissue morphology and physiology to humans and the complex interplay in multiple organs necessitates a dismantled *in vitro* human GBA model. Therefore, we focused on introducing the most advanced 3D gut models and biofabrication methods so far and characterized them by their topographical and geometrical properties. Unfortunately, existing gut models are largely restricted to capturing the typical crypt–villus topography and thus miss capturing the secretory function of the intestine in response to various substances (e.g. microbial metabolites and hormones) related to GBA dysregulation. A gut model with neuroendocrine function is urgently needed and to accelerate advancements of *in vitro* EEC models where proper cell source, material, and fabrication technology should work in harmony (Figure 5).
